# 1,3-Dimethyl-5-methyl­sulfonyl-1*H*-pyrazolo­[4,3-*e*][1,2,4]triazine

**DOI:** 10.1107/S1600536810047264

**Published:** 2010-11-20

**Authors:** Mariusz Mojzych, Zbigniew Karczmarzyk, Waldemar Wysocki

**Affiliations:** a Department of Chemistry, University of Podlasie, ul. 3 Maja 54, 08-110 Siedlce, Poland

## Abstract

In the title compound, C_7_H_9_N_5_O_2_S, the pyrazolo­[4,3-*e*][1,2,4]triazine fused-ring system is essentially planar [maximum deviation = 0.0420 (3) Å]. In the crystal, mol­ecules related by twofold axes are linked into a mol­ecular net *via* inter­molecular C—H⋯O and C—H⋯N hydrogen bonds. π–π inter­actions are observed between the triazine and pyrazole rings of mol­ecules related by the the twofold axis and inversion symmetry with centroid–centroid distances of 3.778 (3) and 3.416 (3) Å, respectively.

## Related literature

For background to sulfones, see: Ingall (1984 ▶). For our work on the development of convenient synthetic approaches for the construction of biologically active heterocycles, see: Karczmarzyk *et al.* (2007 ▶). For related structures, see: Hirata *et al.* (1996 ▶); Rykowski *et al.* (2000 ▶); Cherng-Chyi *et al.* (1994 ▶).
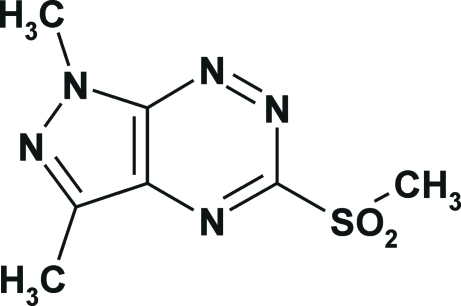


## Experimental

### 

#### Crystal data

C_7_H_9_N_5_O_2_S
*M* *_r_* = 227.25
Monoclinic, 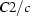

*a* = 17.901 (1) Å
*b* = 8.1268 (7) Å
*c* = 14.203 (3) Å
β = 103.17 (1)°
*V* = 2011.9 (5) Å^3^
*Z* = 8
Mo *K*α radiation
μ = 0.31 mm^−1^
*T* = 293 K
0.40 × 0.30 × 0.10 mm

#### Data collection

Kuma KM-4 four-circle diffractometer
Absorption correction: ψ scan (North *et al.*, 1968 ▶) *T*_min_ = 0.860, *T*_max_ = 0.980
3688 measured reflections
2948 independent reflections
1085 reflections with *I* > 2σ(*I*)
*R*_int_ = 0.064
2 standard reflections every 100 reflections  intensity decay: 1.4%

#### Refinement

*R*[*F* ^2^ > 2σ(*F* ^2^)] = 0.069
*wR*(*F* ^2^) = 0.227
*S* = 1.02
2948 reflections
139 parameters
H-atom parameters constrained
Δρ_max_ = 0.81 e Å^−3^
Δρ_min_ = −0.72 e Å^−3^

### 

Data collection: *KM4B8* (Gałdecki *et al.*, 1996 ▶); cell refinement: *KM4B8*; data reduction: *DATAPROC* (Gałdecki *et al.*, 1995 ▶); program(s) used to solve structure: *SHELXS97* (Sheldrick, 2008 ▶); program(s) used to refine structure: *SHELXL97* (Sheldrick, 2008 ▶); molecular graphics: *ORTEP-3 for Windows* (Farrugia, 1997 ▶); software used to prepare material for publication: *SHELXL97* and *WinGX* (Farrugia, 1999 ▶).

## Supplementary Material

Crystal structure: contains datablocks I, global. DOI: 10.1107/S1600536810047264/pv2349sup1.cif

Structure factors: contains datablocks I. DOI: 10.1107/S1600536810047264/pv2349Isup2.hkl

Additional supplementary materials:  crystallographic information; 3D view; checkCIF report

## Figures and Tables

**Table 1:** Hydrogen-bond geometry (Å, °)

*D*—H⋯*A*	*D*—H	H⋯*A*	*D*⋯*A*	*D*—H⋯*A*
C10—H10*C*⋯O13^i^	0.96	2.51	3.442 (7)	163
C11—H11*B*⋯O14^ii^	0.96	2.42	3.341 (7)	161
C15—H15*A*⋯N2^iii^	0.96	2.59	3.466 (7)	152

Symmetry codes: (i) 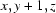; (ii) 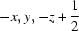; (iii) 
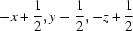
.

**Fig. 1.**
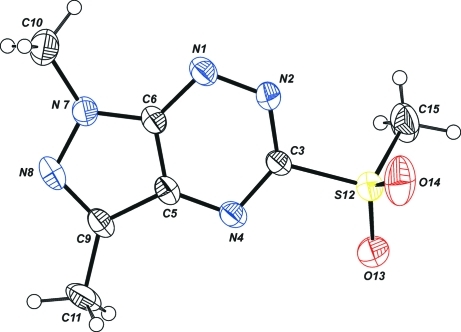
The molecular structure of (I), with atom labels and 50% probability displacement ellipsoids for non-H atoms.

**Fig. 2.**
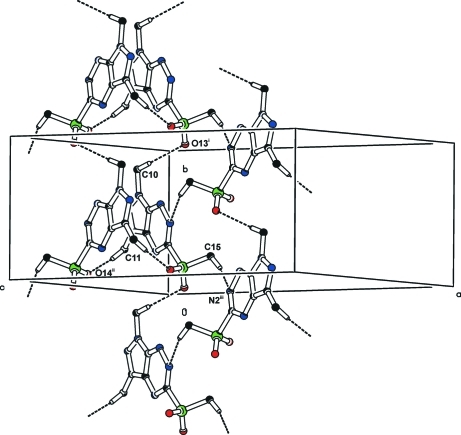
A view of the molecular packing in (I). Dashed lines indicate C—H···X (X = O, N) intermolecular interactions.

## supplementary crystallographic information

### Comment

Sulfones have proven to be valuable synthons for the synthesis of a wide variety of biologically active heterocyclic systems (Ingall, 1984). As an extension of our efforts directed towards the development of convenient synthetic approaches for the construction of biologically active heterocycles (Karczmarzyk *et al.*, 2007), we report herein the crystal and molecular structure of the title compound.

The geometry (bond lengths, angles and planarity) of the title molecule (I) is very similar to those observed in closely related structures (Hirata *et al.*, 1996; Rykowski *et al.*, 2000). In the title molecule, a substitution by methylsulfonyl group in the 1,2,4-triazine ring results in a significant deformation of the endocyclic angles N2—C3—N4 of 130.3 (4)° and C3—N4—C5 of 110.6 (4)°. This effect is caused probably by the strong electron-withdrawing property of SO_2_CH_3_ substituent and has been reported in similar structures (Cherng-Chyi *et al.*, 1994).

In the crystal structure, the molecules related by 2-fold axes are linked into molecular net *via* intermolecular C—H···O and C—H···N hydrogen bonds (Fig. 2 and Tab. 1). In addition, the π-electron systems of the pyrazolo[4,3-*e*][1,2,4]triazine fused rings belonging to inversion- (one side) and 2-fold axis- (other side) related molecules overlap each other, with centroid-to-centroid separation of 3.416 (3) Å between the pyrazole ring at (*x*, *y*, *z*) and triazine ring at (-*x*, 1 - *y*, -*z*), and 3.778 (3) Å between pyrazole ring at (*x*, *y*, *z*) and triazine ring at (-*x*, *y*, 1/2 - *z*). The *π*···*π* distances are 3.2375 (18) and 3.2719 (18) Å, respectively.

### Experimental

To a solution of 2,3-dimethyl-5-methylsulfanyl-1*H*-pyrazolo[4,3-*e*][1,2,4]triazine (1 mmol) in benzene (20 ml), water (30 ml), potassium manganate (VII) (3 mmol), catalitic amounts of tetrabuthylammonium bromide (0.2 mmol) and acetic acid (1.5 ml) were added. The reaction mixture was stirred at room temperature for 1 h. A saturated solution of Na_2_S_2_O_5 _in water was then added to the mixture until the purple colour disappeared. The organic layer was separated and the aqueos phase was extracted with benzene (3x10 ml). The combined organic extracts were dried over anhydrous MgSO_4 _and concentrated *in vacuo*. The residue was purified by column chromatography on silica gel (eluent: chloroform) to afford the title compound as a yellow solid. Crystals suitable for X-ray diffraction analysis were grown by slow evaporation of an ethanol solution.

### Refinement

The H atoms were positioned geometrically and treated as riding on their C atoms, with C—H distances of 0.96 Å and were included in the refinement with *U*_iso_(H) = 1.5*U*_eq_(C).

### Crystal data

**Table:**

C_7_H_9_N_5_O_2_S	*F*(000) = 944
*M**_r_* = 227.25	*D*_x_ = 1.500 Mg m^−^^3^
Monoclinic, *C*2/*c*	Melting point: 444 K
Hall symbol: -C 2yc	Mo *K*α radiation, λ = 0.71073 Å
*a* = 17.901 (1) Å	Cell parameters from 25 reflections
*b* = 8.1268 (7) Å	θ = 4.4–25.2°
*c* = 14.203 (3) Å	µ = 0.31 mm^−^^1^
β = 103.17 (1)°	*T* = 293 K
*V* = 2011.9 (5) Å^3^	Prism, colourless
*Z* = 8	0.40 × 0.30 × 0.10 mm

### Data collection

**Table:**

Kuma KM-4 four-circle diffractometer	1085 reflections with *I* > 2σ(*I*)
Radiation source: fine-focus sealed tube	*R*_int_ = 0.064
graphite	θ_max_ = 30.1°, θ_min_ = 2.3°
ω–2θ scans	*h* = −25→24
Absorption correction: ψ scan (North *et al.*, 1968)	*k* = −1→11
*T*_min_ = 0.860, *T*_max_ = 0.980	*l* = −1→19
3688 measured reflections	2 standard reflections every 100 reflections
2948 independent reflections	intensity decay: 1.4%

### Refinement

**Table:**

Refinement on *F*^2^	Primary atom site location: structure-invariant direct methods
Least-squares matrix: full	Secondary atom site location: difference Fourier map
*R*[*F*^2^ > 2σ(*F*^2^)] = 0.069	Hydrogen site location: inferred from neighbouring sites
*wR*(*F*^2^) = 0.227	H-atom parameters constrained
*S* = 1.02	*w* = 1/[σ^2^(*F*_o_^2^) + (0.1*P*)^2^] where *P* = (*F*_o_^2^ + 2*F*_c_^2^)/3
2948 reflections	(Δ/σ)_max_ < 0.001
139 parameters	Δρ_max_ = 0.81 e Å^−^^3^
0 restraints	Δρ_min_ = −0.72 e Å^−^^3^

### Special details

**Table:**

Experimental. Yield: 95% and m.p. 444 K. ^1^H NMR (CDCl_3_) *δ*: 2.77 (*s*, 3H), 3.57 (*s*, 3H), 4.39 (*s*, 3H); IR (KBr,ν, cm^-1^): 2920, 1330, 1120; MS (*m/z*, %): 227 (8) [*M*^+^], 199 (32), 120 (21), 95 (51), 79 (94), 67 (28), 52 (100). Analysis calculated for C_7_H_9_N_5_O_2_S: C 37.00, H 3.99, N 30.82%; found: C 37.01, H 3.85, N 30.76%.
Geometry. All esds (except the esd in the dihedral angle between two l.s. planes) are estimated using the full covariance matrix. The cell esds are taken into account individually in the estimation of esds in distances, angles and torsion angles; correlations between esds in cell parameters are only used when they are defined by crystal symmetry. An approximate (isotropic) treatment of cell esds is used for estimating esds involving l.s. planes.
Refinement. Refinement of F^2^ against ALL reflections. The weighted R-factor wR and goodness of fit S are based on F^2^, conventional R-factors R are based on F, with F set to zero for negative F^2^. The threshold expression of F^2^ > 2sigma(F^2^) is used only for calculating R-factors(gt) etc. and is not relevant to the choice of reflections for refinement. R-factors based on F^2^ are statistically about twice as large as those based on F, and R- factors based on ALL data will be even larger.

### Fractional atomic coordinates and isotropic or equivalent isotropic displacement parameters (Å^2^)

**Table:**

	*x*	*y*	*z*	*U*_iso_*/*U*_eq_	
S12	0.15935 (7)	0.15917 (15)	0.19815 (10)	0.0433 (4)	
O13	0.1310 (2)	0.0271 (5)	0.1348 (3)	0.0748 (13)	
O14	0.1737 (2)	0.1313 (5)	0.3001 (3)	0.0765 (13)	
N1	0.0756 (2)	0.6040 (5)	0.1881 (3)	0.0412 (10)	
N2	0.1192 (2)	0.4699 (5)	0.2062 (3)	0.0373 (9)	
N4	0.02186 (19)	0.2860 (5)	0.1191 (3)	0.0361 (9)	
N7	−0.0502 (2)	0.6833 (5)	0.0994 (3)	0.0388 (9)	
N8	−0.1138 (2)	0.6061 (5)	0.0483 (3)	0.0435 (10)	
C3	0.0915 (2)	0.3259 (5)	0.1696 (3)	0.0317 (9)	
C5	−0.0227 (2)	0.4200 (5)	0.1003 (3)	0.0336 (10)	
C6	0.0051 (2)	0.5751 (5)	0.1322 (3)	0.0332 (10)	
C9	−0.0993 (2)	0.4447 (6)	0.0469 (3)	0.0377 (11)	
C10	−0.0494 (3)	0.8619 (6)	0.1113 (4)	0.0586 (15)	
H10A	−0.0770	0.8909	0.1594	0.088*	
H10B	−0.0732	0.9128	0.0509	0.088*	
H10C	0.0027	0.8995	0.1315	0.088*	
C11	−0.1567 (3)	0.3236 (6)	−0.0021 (4)	0.0567 (14)	
H11A	−0.2011	0.3804	−0.0381	0.085*	
H11B	−0.1712	0.2539	0.0453	0.085*	
H11C	−0.1350	0.2579	−0.0454	0.085*	
C15	0.2416 (3)	0.2357 (8)	0.1689 (5)	0.0646 (17)	
H15A	0.2807	0.1526	0.1803	0.097*	
H15B	0.2593	0.3302	0.2083	0.097*	
H15C	0.2303	0.2669	0.1020	0.097*	

### Atomic displacement parameters (Å^2^)

**Table:**

	*U*^11^	*U*^22^	*U*^33^	*U*^12^	*U*^13^	*U*^23^
S12	0.0358 (6)	0.0407 (7)	0.0504 (7)	0.0015 (6)	0.0035 (5)	0.0050 (6)
O13	0.058 (2)	0.044 (2)	0.108 (3)	0.0052 (19)	−0.011 (2)	−0.022 (2)
O14	0.075 (3)	0.096 (3)	0.057 (2)	0.030 (2)	0.012 (2)	0.036 (2)
N1	0.0313 (19)	0.047 (2)	0.042 (2)	−0.0005 (18)	0.0021 (16)	−0.0085 (19)
N2	0.0316 (18)	0.038 (2)	0.039 (2)	0.0034 (17)	0.0013 (15)	0.0006 (18)
N4	0.0315 (18)	0.040 (2)	0.035 (2)	−0.0049 (16)	0.0025 (16)	0.0024 (17)
N7	0.0339 (19)	0.040 (2)	0.040 (2)	0.0048 (18)	0.0033 (16)	−0.0033 (18)
N8	0.0247 (18)	0.063 (3)	0.041 (2)	0.0028 (18)	0.0028 (16)	0.000 (2)
C3	0.0264 (18)	0.036 (2)	0.031 (2)	−0.0035 (19)	0.0035 (16)	0.001 (2)
C5	0.0250 (19)	0.045 (3)	0.030 (2)	0.000 (2)	0.0058 (16)	0.003 (2)
C6	0.027 (2)	0.041 (3)	0.030 (2)	0.0020 (19)	0.0049 (16)	−0.006 (2)
C9	0.025 (2)	0.051 (3)	0.035 (3)	−0.004 (2)	0.0017 (18)	0.007 (2)
C10	0.057 (3)	0.052 (3)	0.061 (4)	0.014 (3)	0.000 (3)	−0.003 (3)
C11	0.036 (2)	0.065 (3)	0.062 (3)	−0.022 (3)	−0.005 (2)	0.006 (3)
C15	0.035 (3)	0.073 (4)	0.089 (4)	0.009 (3)	0.020 (3)	0.023 (3)

### Geometric parameters (Å, °)

**Table:**

S12—O13	1.418 (4)	C5—C6	1.393 (6)
S12—O14	1.430 (4)	C5—C9	1.423 (6)
S12—C15	1.733 (5)	C9—C11	1.476 (6)
S12—C3	1.803 (4)	C10—H10A	0.9600
N1—N2	1.331 (5)	C10—H10B	0.9600
N1—C6	1.350 (5)	C10—H10C	0.9600
N2—C3	1.330 (5)	C11—H11A	0.9600
N4—C3	1.329 (5)	C11—H11B	0.9600
N4—C5	1.340 (5)	C11—H11C	0.9600
N7—C6	1.326 (5)	C15—H15A	0.9600
N7—N8	1.357 (5)	C15—H15B	0.9600
N7—C10	1.461 (6)	C15—H15C	0.9600
N8—C9	1.338 (6)		
			
O13—S12—O14	118.6 (3)	N8—C9—C5	107.3 (4)
O13—S12—C15	108.7 (3)	N8—C9—C11	123.0 (4)
O14—S12—C15	109.4 (3)	C5—C9—C11	129.8 (4)
O13—S12—C3	107.5 (2)	N7—C10—H10A	109.5
O14—S12—C3	107.6 (2)	N7—C10—H10B	109.5
C15—S12—C3	104.0 (2)	H10A—C10—H10B	109.5
N2—N1—C6	113.5 (4)	N7—C10—H10C	109.5
N1—N2—C3	119.7 (3)	H10A—C10—H10C	109.5
C3—N4—C5	110.6 (4)	H10B—C10—H10C	109.5
C6—N7—N8	110.5 (4)	C9—C11—H11A	109.5
C6—N7—C10	129.1 (4)	C9—C11—H11B	109.5
N8—N7—C10	120.4 (4)	H11A—C11—H11B	109.5
C9—N8—N7	108.6 (3)	C9—C11—H11C	109.5
N4—C3—N2	130.3 (4)	H11A—C11—H11C	109.5
N4—C3—S12	116.0 (3)	H11B—C11—H11C	109.5
N2—C3—S12	113.6 (3)	S12—C15—H15A	109.5
N4—C5—C6	121.3 (4)	S12—C15—H15B	109.5
N4—C5—C9	132.7 (4)	H15A—C15—H15B	109.5
C6—C5—C9	106.0 (4)	S12—C15—H15C	109.5
N7—C6—N1	127.9 (4)	H15A—C15—H15C	109.5
N7—C6—C5	107.7 (4)	H15B—C15—H15C	109.5
N1—C6—C5	124.4 (4)		
			
C6—N1—N2—C3	0.2 (6)	C10—N7—C6—N1	2.4 (8)
C6—N7—N8—C9	−0.8 (5)	N8—N7—C6—C5	1.0 (5)
C10—N7—N8—C9	179.0 (4)	C10—N7—C6—C5	−178.8 (5)
C5—N4—C3—N2	4.2 (6)	N2—N1—C6—N7	−177.7 (4)
C5—N4—C3—S12	−178.4 (3)	N2—N1—C6—C5	3.7 (6)
N1—N2—C3—N4	−4.5 (7)	N4—C5—C6—N7	177.2 (4)
N1—N2—C3—S12	178.1 (3)	C9—C5—C6—N7	−0.8 (5)
O13—S12—C3—N4	17.9 (4)	N4—C5—C6—N1	−3.9 (7)
O14—S12—C3—N4	−110.9 (4)	C9—C5—C6—N1	178.1 (4)
C15—S12—C3—N4	133.0 (4)	N7—N8—C9—C5	0.2 (5)
O13—S12—C3—N2	−164.3 (3)	N7—N8—C9—C11	179.5 (4)
O14—S12—C3—N2	66.9 (4)	N4—C5—C9—N8	−177.3 (4)
C15—S12—C3—N2	−49.2 (4)	C6—C5—C9—N8	0.3 (5)
C3—N4—C5—C6	0.0 (6)	N4—C5—C9—C11	3.5 (8)
C3—N4—C5—C9	177.3 (4)	C6—C5—C9—C11	−178.9 (5)
N8—N7—C6—N1	−177.9 (4)		

### Hydrogen-bond geometry (Å, °)

**Table:**

*D*—H···*A*	*D*—H	H···*A*	*D*···*A*	*D*—H···*A*
C10—H10C···O13^i^	0.96	2.51	3.442 (7)	163
C11—H11B···O14^ii^	0.96	2.42	3.341 (7)	161
C15—H15A···N2^iii^	0.96	2.59	3.466 (7)	152
Cg(pyrazole)—···.Cg(triazine)^ii^	.	.	3.778 (3)	.
Cg(pyrazole)—···.Cg(triazine)^iv^	.	.	3.416 (3)	.

Symmetry codes: (i) *x*, *y*+1, *z*; (ii) −*x*, *y*, −*z*+1/2; (iii) −*x*+1/2, *y*−1/2, −*z*+1/2; (iv) *x*+1/2, *y*+3/2, *z*.
